# Full participation in harm reduction programmes is associated with decreased risk for human immunodeficiency virus and hepatitis C virus: evidence from the Amsterdam Cohort Studies among drug users

**DOI:** 10.1111/j.1360-0443.2007.01912.x

**Published:** 2007-09

**Authors:** Charlotte Van Den Berg, Colette Smit, Giel Van Brussel, Roel Coutinho, Maria Prins

**Affiliations:** 1 Department of Human Retrovirology, Center for Infection and Immunity Amsterdam (CINIMA), Academic Medical Center Amsterdam, the Netherlands; 2 Cluster Infectious Diseases, Department of Research, Amsterdam Health Service Amsterdam, the Netherlands; 3 Cluster Social and Mental Healthcare, Amsterdam Health Service Amsterdam, the Netherlands; 4 National Institute for Public Health and the Environment, Center for Infectious Disease Control Bilthoven, the Netherlands; 5 Department of Internal Medicine, Division of Infectious Diseases, Tropical Medicine and AIDS, CINIMA, Academic Medical Center Amsterdam, the Netherlands

**Keywords:** Harm reduction, hepatitis C virus, HIV, injecting drug use, methadone, needle exchange programmes

## Abstract

**Objectives:**

To investigate the impact of harm-reduction programmes on HIV and hepatitis C virus (HCV) incidence among ever-injecting drug users (DU) from the Amsterdam Cohort Studies (ACS).

**Methods:**

The association between use of harm reduction and seroconversion for human immunodeficiency virus (HIV) and/or hepatitis C virus (HCV) was evaluated using Poisson regression. A total of 714 DU were at risk for HIV and/or HCV during follow-up. Harm reduction was measured by combining its two most important components—methadone dose and needle exchange programme (NEP) use—and looking at five categories of participation, ranging from no participation (no methadone in the past 6 months, injecting drug use in the past 6 months and no use of NEP) to full participation (≥ 60 mg methadone/day and no current injecting or ≥ 60 mg methadone/day and current injecting but all needles exchanged).

**Results:**

Methadone dose or NEP use alone were not associated significantly with HIV or HCV seroconversion. However, with combination of these variables and after correction for possibly confounding variables, we found that full participation in a harm reduction programme (HRP) was associated with a lower risk of HIV and HCV infection in ever-injecting drug users (DU), compared to no participation [incidence rate ratio 0.43 (95% CI 0.21–0.87) and 0.36 (95% CI 0.13–1.03), respectively].

**Conclusions:**

In conclusion, we found that full participation in HRP was associated with a lower incidence of HCV and HIV infection in ever-injecting DU, indicating that combined prevention measures—but not the use of NEP or methadone alone—might contribute to the reduction of the spread of these infections.

## INTRODUCTION

Injecting drug users (DU) are at high risk from blood-borne infections, including human immunodeficency virus (HIV) and hepatitis C virus (HCV), through the sharing of needles and injection equipment [1]. Various approaches to deal with the consequences of hard drugs have been taken; some countries aim to ban illicit drug use completely, whereas the Netherlands and others take a harm reduction approach. This harm reduction approach may have had a major impact on the HIV and HCV epidemic. The ultimate goal of harm reduction is to stop DU from using drugs, but until this is possible the policy is to minimize the damage DU inflict upon themselves and the society at large. Diverse programmes (with a low, medium or high threshold) [2] started in the Netherlands at theend of the 1970s, providing methadone in combination with social–medical care and needle-exchange facilities. They have no waiting lists and are relatively easy to enter and re-enter. Ongoing drug use during participation is tolerated in low- and medium-threshold programmes. Low-threshold programmes have been operated since 1982 by the Amsterdam Health Service. For clients who have regulated their drug use, methadone can be prescribed in a medium-threshold programme via their general practitioner. Clients who are willing to detoxify can receive methadone in a high-threshold programme through an out-patient addiction clinic. Circulation between the different programmes is permitted and ‘promotion’ to higher-threshold programmes is encouraged. With the harm reduction approach, the Amsterdam methadone programmes reached an estimated 2700 of the 3500–4000 opiate users in Amsterdam [3]. All services are free of charge for residents of the Netherlands.

The effects of methadone provision or needle exchange programmes (NEP) separately on HIV incidence have been examined, with conflicting results [4,5]. Very few studies describe the effect of either programme on HCV incidence, although declining prevalence of HCV was reported after the introduction of NEP [6].

The Amsterdam Cohort Study (ACS) among drug users comprises a large group of DU who are tested prospectively for HIV. We tested their stored sera for HCV, retrospectively, and therefore had the unique opportunity to document the effect of harm reduction on the incidence of both HIV and HCV over a long time period [7–9].

## MATERIALS AND METHODS

### Study population and design

The Amsterdam Cohort Study (ACS) among DU is an open, prospective cohort study initiated to investigate the prevalence, incidence and risk factors of infections with HIV-1 and other blood-borne and/or sexually transmitted infections, as well as the effects of interventions [10]. It has collected detailed information on participation in harm reduction programmes (HRPs). The DU cohort was initiated in 1985; recruitment is ongoing and in recent years has been directed in particular towards young DU.

ACS participation is voluntary, and informed consent is obtained for every participant at intake. ACS participants visit the Amsterdam Health Service every 4–6 months. At intake and every visit, they give blood for HIV testing and storage; they also complete a standardized questionnaire about their health, drug use and sexual risk behaviour and socio-demographic situation. At intake, questions about current behaviour refer to the preceding 6 months and/or to the period since 1980 or since the start of regular use of hard drugs (i.e. heroin, cocaine, amphetamines and/or methadone at least three times per week). At follow-up visits, questions refer to the time between the present and the preceding visit.

### Laboratory methods

All ACS participants since 1985 (*n* = 1640) were tested prospectively for HIV antibodies by enzyme-linked immunosorbent assays (ELISA). All participants with at least two visits between December 1985 and November 2005 (*n* = 1276) were tested retrospectively for HCV antibodies, using the first sample available in each case. Third-generation ELISA tests were used to detect HCV antibodies (AxSym HCV version 3.0; Abbott, Wiesbaden, Germany). Individuals who were HCV-negative at ACS entry were tested for HCV antibodies at their most recent ACS visit. On finding HCV seroconversion (defined as the presence of HCV antibodies in a previously seronegative individual), we tested samples taken between these two visits to indicate the seroconversion interval.

### Statistical analyses

HIV- and/or HCV-negative ever-injecting drug users entered the risk set at study entry or at their start of injecting drug use during follow-up, and were followed-up until seroconversion for, respectively, HIV or HCV, or until end of follow up, ultimately at 1 November 2005. The date of HCV or HIV seroconversion was estimated as the midpoint between the last seronegative and the first seropositive ACS visit. Poisson regression was used to determine the effect of harm reduction on HCV and HIV incidence. Incidence rates and incidence rate ratios (IRR) with their corresponding 95% confidence intervals (95% CI) were calculated. We evaluated the potential confounding effect of all variables listed below and evaluated interaction between variables included in the final model. Multivariate models were built using forward-stepwise techniques, and variables with a univariate *P*-value ≤ 0.10 were considered as potential independent determinants. All variables subject to change were treated as time-dependent variables. A *P*-value ≤ 0.05 was considered statistically significant.

To study the impact of harm reduction on HIV and HCV seroconversion, we combined injecting drug use, use of NEP and methadone dosage into one variable with five categories (Table 1). Because higher doses of methadone are more effective than lower doses in lowering the prevalence of injecting drug use risk behaviour, we considered ≥ 60 mg methadone per day an adequate minimum dosage for opioid replacement therapy and used that dose as cut-off value for our definition of adequate harm reduction [11–13].

**Table 1:** Definition of five levels of harm reduction used to evaluate the effect of harm reduction on HIV and HCV incidence in the Amsterdam Cohort Studies.

No harm reduction	No methadone in the past 6 months, injecting drug use in the past 6 months, and no use of NEP
Incomplete harm reduction	Any dose of methadone daily in the past 6 months, injecting drug use in the past 6 months and irregular* or no use of NEP; OR 0–59 mg methadone daily in the past 6 months, injecting drug use in the past 6 months, and always use† of NEP
Full harm reduction	≥ 60 mg methadone daily in the past 6 months and no injecting drug use in the past 6 months; OR ≥ 60 mg methadone daily, injecting drug use in the past 6 months, and always use† of NEP
Limited dependence on harm reduction	1–59 mg methadone daily in the past 6 months and no injecting drug use in the past 6 months
No dependence on harm reduction	No methadone in the past 6 months and no injecting drug use in the past 6 months

* Irregular use of NEP = 1–99% of needles used in the past 6 months obtained via NEP.

† Always use of NEP = 100% of needles used in past 6 months obtained via NEP.

General characteristics of persons evaluated included sex, nationality, age, HIV status for HCV as outcome, HCV status for HIV as outcome, HIV status of the steady partner, homelessness and hospitalization. The drug use variables included current injecting (yes or no), frequency of injecting, main type of drug injected, time elapsed since start of injecting drug use, frequency of non-injecting drug use and type of drug used mainly as non-injecting drug.

## RESULTS

### General characteristics

In total, 1640 DU were enrolled in the ACS; 1276 DU had at least two visits. DU with more than one visit were older [median 31.4 (interquartile range (IQR) 31.0–31.8) years versus 28.7 (IQR 28.1–29.4) years], more often male (63.9% versus 56.9%), more often of Dutch nationality (74.5% versus 60.2%) and more often HIV-positive (20.6% versus 16.2%) when compared to DU with only one visit to the ACS.

A total of 952 DU were so-called ever-injecting DU: DU who had ever injected drugs before ACS entry (*n* = 905) or who started injecting drugs during follow-up (*n* = 47). Of these ever-injecting DU, 714 were HIV- and/or HCV-negative at study entry and were at risk for HIV and/or HCV during follow-up. One hundred and sixty-four DU (22.9%) were negative for both infections at study entry, 546 DU (76.5%) were HIV-negative and HCV-positive and four DU (0.6%) were HCV-negative and HIV-positive. The HIV prevalence among HCV-negative DU was 2.4% at entry, while the HCV prevalence among HIV-negative DU was much higher (76.2%). The DU included were mainly of Dutch nationality and mainly male (Table 2).

**Table 2:** General characteristics at entry and during follow-up of 710 HIV-negative and 168 HCV-negative ever-injecting DU included in HIV and HCV analyses, respectively.

	HIV		HCV	
*At entry*				
HIV/HCV infection (*n* at risk)	710	%	168	%
Prevalence HIV infection at entry risk set	–		4	2.4
Prevalence HCV infection at entry risk set	541	76.2	–	
Overall HIV incidence (per 100 PY)	1.65			
Overall HCV incidence (per 100 PY)			6.78	
General characteristics				
Steady partner at entry	333	46.9	77	45.8
Median age at entry risk set (years; IQR)	30.0 (27.0–36.0)		29.0 (25.0–33.0)	
Female	274	38.6	56	33.3
Dutch nationality	526	74.1	147	87.5
Western European ethnicity	602	84.8	139	82.7
Injecting drug use				
Median time since start injecting (years; IQR)	7.21 (3.04–12.1)		2.43 (0.06–7.16)	
Injecting in the past 6 months	524	73.8	100	59.5
Among recent injectors injecting more than once a week	429	82.3	53	54.6
Main drug injected				
Heroin	94	17.9	33	33.0
Cocaine	77	14.7	14	14.0
Speedball (i.e. combination of heroin and cocaine) other	271	51.7	37	37.0
	82	15.6	16	16.0
Non-injecting drug use				
Non-injecting drug use in the past 6 months	497	70.0	149	88.7
Frequency of non-injecting drug use				
Once or more times daily	190	38.2	77	51.7
Once or more times weekly, but less than once or more times daily	188	37.8	61	41.0
Less than weekly	119	23.9	11	7.4
Main non-injecting drug use at entry				
Heroin	239	48.2	66	44.2
Cocaine	215	43.3	73	49.0
Other	42	8.5	10	6.7
*Follow-up*				
Median number of visits at risk (IQR)	17 (8–29)		15 (8–28)	
Median number of PY (IQR)	8.13 (4.25–13.0)		3.56 (1.15–7.91)	
Median number of days between follow up visits (IQR)	128 (118–168)		128 (119–166)	

PY = person years; IQR = interquartile range.

HIV-negative DU had a longer median time since starting injection than HCV-negative DU (respectively, 7.4 and 2.4 years). Furthermore, the proportion of DU who had recently injected (i.e. in the past 6 months before ACS entry) was larger for the HIV-negative DU than for HCV-negative DU. HIV-negative DU injected more often than HCV-negative DU, and HCV-negative DU used non-injecting drugs more often than their HIV-negative counterparts (Table 2). The median follow-up time was 3.56 years (IQR 1.15–7.91 years) for DU at risk for HCV and 8.13 years (IQR 4.25–13.0 years) for DU at risk for HIV.

Under study, 90 of 710 DU at risk for HIV seroconverted and 58 of 168 at risk for HCV. The median duration of the HIV and HCV seroconversion interval between visits was 4.0 months (IQR 3.7–6.0 months) and 4.0 months (IQR 3.7–5.1 months), respectively. The HIV incidence ranged from 8.5 per 100 person-years (PY) in the late 1980s to approximately 0 in the most recent years, whereas HCV incidence was very high in the late 1980s (27.5 per 100 PY) and declined to around two per 100 PY in more recent years [14].

### Effect of harm reduction participation on HIV and HCV incidence

When evaluating the separate effects on HIV and HCV seroconversion of methadone dose or NEP we found that having any prescribed dose of methadone was associated with lower incidence rates of HIV and HCV infection, but not to a statistically significant degree (*P* = 0.084 and *P* = 0.21, respectively). Use of NEP was associated with a higher risk of HIV and HCV seroconversion but, with restriction of this analysis to injecting drug use in the preceding 6 months, the IRR changed towards one and no longer reached statistical significance (data not shown). However, when methadone dose and NEP were combined as described in Table 1, full participation in an HRP was associated with a two- to threefold reduction in the risk of HIV seroconversion and with a six- to sevenfold reduction in the risk of HCV seroconversion (Table 3).

**Table 3:** Univariate associations between general characteristics, drug use characteristics, sexual risk behaviour characteristics and HIV and HCV seroconversion among DU in the ACS.

	HIV						HCV					
		
	Incidence (/100 PY)	sc	PY	IRR	95% CI	P-value	Incidence (/100 PY)	sc	PY	IRR	95% CI	P-value
*Harm reduction*												
Level of harm reduction (definitions described in Table 1)												
No harm reduction	3.80	18	473.6	1		< 0.001	23.16	11	47.5	1		< 0.001
Incomplete harm reduction	2.80	46	1640.8	0.74	(0.43–1.27)		24.12	34	141.0	1.04	(0.53–2.05)	
Full harm reduction	1.22	18	1475.9	0.32	(0.17–0.62)		3.47	6	173.0	0.15	(0.056–0.40)	
Limited dependence	0.13	1	758.1	0.035	(0.005–0.26)		0.57	1	174.9	0.024	(0.003–0.19)	
No dependence	0.57	6	1048.4	0.15	(0.060–0.38)		1.64	5	305.2	0.071	(0.025–0.20)	
Methadone dosage												
0 mg	2.16	44	2036.9	1		0.084	8.34	34	407.7	1		0.21
0–60 mg	1.37	21	1531.8	0.63	(0.38–1.07)		4.87	11	226.1	0.58	(0.30–1.15)	
≥ 60 mg	1.33	25	1880.6	0.62	(0.38–1.01)		5.67	12	211.8	0.68	(0.35–1.31)	
Needle exchange programme (% of needles obtained via)												
No recent injecting	0.38	10	2633.8	1		< 0.001	1.60	10	623.4	1		< 0.001
0%	3.07	26	847.3	8.08	(3.90–16.8)		19.66	18	91.6	12.3	(5.66–26.6)	
1–99%	2.30	8	347.8	6.05	(2.39–15.4)		30.56	11	36.0	19.0	(8.09–44.9)	
100%	2.91	46	1578.8	7.67	(3.87–15.2)		19.10	19	99.5	11.9	(5.54–25.6)	
Change in methadone dosage compared to previous visit												
No change	1.34	39	2917.1	1		0.11	3.73	16	428.4	1		< 0.001
Increase	1.82	18	991.5	1.36	(0.78–2.37)		3.64	5	137.2	0.98	(0.36–2.66)	
Decrease	1.67	13	778.5	1.25	(0.67–2.34)		5.83	6	102.8	1.56	(0.61–3.99)	
Unknown	2.56	20	781.2	1.99	(1.12–3.83)		16.51	31	187.8	4.42	(2.42–8.08)	
*General characteristics*												
Sex												
Male	1.57	53	3384.1	1		0.62	5.65	32	566.0	1		0.085
Female	1.78	37	2084.3	1.13	(0.74–1.72)		8.96	26	290.2	1.58	(0.94–2.66)	
Age (per 10 years increase)				0.52	(0.39–0.69)	< 0.001				0.46	(0.32–0.65)	< 0.001
Homelessness												
No	1.58	82	5176.5	1		0.18	6.5	52	799.6	1		0.29
Yes	2.74	8	291.9	1.71	(0.83–3.54)		10.6	6	56.7	1.63	(0.70–3.79)	
Hospitalization in preceding 6 months												
No	1.56	80	5124.1	1.86	(0.96–3.59)	0.09	6.85	56	817.1	0.75	(0.18–3.05)	0.67
Yes	2.90	10	344.3	1			5.11	2	39.2	1		
HCV/HIV status at visit												
Negative	1.13	10	888.5	1		0.41	6.13	51	831.5	1		0.0055
Positive	1.73	79	4553.6	1.54	(0.80–2.98)		34.82	5	14.4	5.68	(2.27–14.2)	
Acute infection in previous 6 months	4.64	1	21.6	4.12	(0.53–32.2)		19.18	2	10.4	3.12	(0.76–12.8)	
*Drug use variables*												
Injecting in past 6 months												
Yes	2.83	80	2831.1	7.45	(3.86–14.4)	< 0.001	20.74	48	231.5	12.9	(6.54–25.6)	< 0.001
No	0.38	10	2633.8	1			1.60	10	623.4	1		
Borrowing of needles												
No recent injecting	0.38	10	2633.8	1		< 0.001	1.60	10	623.4	1		< 0.001
Recent injecting, no borrowing	2.71	54	1996.3	7.12	(3.63–14.0)		14.51	23	158.5	9.05	(4.31–19.0)	
Recent injecting, borrowing 1–9 times	3.30	12	363.6	8.69	(3.76–20.1)		48.11	14	29.1	30.0	(13.3–67.5)	
Recent injecting, borrowing ≥ 10 times	2.17	1	46.2	5.71	(0.73–44.6)		23.78	2	8.41	14.8	(3.25–67.7)	
Frequency of injecting drug use in previous 6 months												
No injecting drug use in previous 6 months	0.38	10	2633.8	1		< 0.001	1.60	10	623.4	1		< 0.001
≥ 2 times/day	3.66	32	874.6	9.63	(4.74–19.6)		34.72	17	49.0	21.6	(9.91–47.3)	
Once/day	2.71	3	110.6	7.15	(1.97–26.0)		25.45	1	3.93	15.8	(2.03–123.8)	
≥ 2 times/week	3.05	29	949.6	8.04	(3.92–16.5)		28.83	19	65.9	18.0	(8.36–38.7)	
Once a week	0.00	0	147.5	0			8.23	1	12.2	5.13	(0.66–40.1)	
≥ 2 times/month	2.15	5	232.1	5.67	(1.94–16.6)		8.13	3	36.9	5.07	(1.40–18.4)	
Once/month	3.92	4	102.0	10.33	(3.24–33.0)		15.19	2	13.2	9.47	(2.07–43.2)	
Less frequent	1.53	6	393.2	4.02	(1.46–11.1)		8.36	4	47.9	5.21	(1.63–16.6)	
Main drug injected in previous 6 months												
No injecting drug use in previous 6 months	0.38	10	2633.8	1		< 0.001	1.60	10	623.4	1		< 0.001
Heroin	2.26	13	574.2	5.96	(2.61–13.6)		19.62	9	45.9	12.2	(4.97–30.1)	
Cocaine	1.81	8	442.7	4.76	(1.88–12.1)		24.31	10	41.1	15.2	(6.31–36.4)	
Speedball	3.41	48	1408.6	8.97	(4.54–17.7)		18.30	21	114.7	11.4	(5.37–24.2)	
Amphetamines	1.87	4	213.9	4.92	(1.54–15.7)					7.45	(1.63–34.0)	
Methadone	3.02	4	132.6	7.95	(2.49–25.3)					21.3	(5.87–77.5)	
Other	4.94	3	60.8	13.0	(3.58–47.2)		26.92	8	29.7	44.5	(12.2–161.6)	
Time since start injection drug use (years)				0.93	(0.91–0.96)	< 0.001				0.80	(0.74–0.86)	< 0.001
*Sexual risk behaviour*												
Heterosexual risk behaviour in previous 6 months												
No	1.59	52	3270.9	1		0.87	7.57	36	475.9	1		0.59
Yes	1.73	38	2192.5	1.09	(0.72–1.66)		5.79	22	380.0	0.77	(0.45–1.30)	
HIV status of steady partner												
No steady partner	1.63	67	4122.5	1		0.0013	7.83	53	676.7	1		0.020
HIV-positive	6.90	10	145.0	4.24	(2.18–8.25)		10.71	2	18.7	1.37	(0.33–5.61)	
HIV-negative	1.14	11	963.7	0.70	(0.37–1.33)		2.62	3	114.6	0.33	(0.10–1.07)	
Unknown HIV status	0.98	2	203.8	0.60	(0.15–2.46)		0.00	0	34.0	0		

Abbreviations: sc = seroconversion, PY = person years, IRR = incidence rate ratio, 95% CI = 95% confidence interval.

In univariate analysis the following variables were also associated with a higher risk of HIV or HCV: injecting drug use in the past 6 months, borrowing needles in the past 6 months, more recent onset of injecting drug use, a higher frequency of injecting drugs, mainly injecting speedball, younger age and having an HIV-positive steady partner. A change in methadone dosage in the past 6 months was associated with a higher risk for HCV seroconversion but not HIV seroconversion. DU who were chronically HIV-infected or had an acute HIV infection in the 6 months preceding the visit were at increased risk for HCV seroconversion (Table 3).

In multivariate analysis we found that after correcting for having an HIV-positive steady partner and a smaller number of years since starting injection (both factors being associated independently with HIV seroconversion), the combined harm reduction variable remained associated independently with HIV seroconversion (Table 4). That is, DU fully participating in HRPs were at a decreased risk of HIV seroconversion compared to DU not participating fully in an HRP (IRR 0.43, 95% CI 0.21–0.87).

**Table 4:** Multivariate analysis of the effect of participation in harm reduction programmes on HIV and HCV seroconversion.

	HIV			HCV		
		
	IRR	95% CI	P-value	IRR	95% CI	P-value
Level of harm reduction (definitions described in Table 1)						
No harm reduction	1		< 0.001	1		< 0.001
Incomplete harm reduction	0.87	0.50–1.52		1.17	0.59–2.31	
Full harm reduction	0.43	0.21–0.87		0.36	0.13–1.03	
Limited dependence on harm reduction	0.046	0.006–0.35		0.044	0.006–0.35	
No dependence on harm reduction	0.20	0.078–0.50		0.13	0.044–0.40	
Time since start injection drug use (per year)	0.95	0.92–0.98	< 0.001	0.87	0.81–0.93	< 0.001
HIV status of steady partner						
No steady partner	1		0.004	1		0.026
HIV-positive steady partner	4.53	2.23–9.21		3.49	0.84–14.5	
HIV-negative steady partner	0.82	0.43–1.57		0.42	0.13–1.37	
Steady partner with unknown HIV status	0.75	0.18–3.06				

IRR = incidence rate ratio, 95% CI = 95% confidence interval.

In multivariate analysis for HCV, we found that with correction for time elapsed since start of injecting, DU participating fully in an HRP were at decreased risk of HCV seroconversion compared to DU not participating in an HRP (IRR 0.36, 95% CI 0.13–1.03). As with HIV, DU who recently started injecting drug use were at increased risk of HCV seroconversion. The effect of HIV status of the steady partner on HCV incidence had the same direction as its effect on HIV incidence (Table 4).

In sensitivity analyses, we found that the effects of harm reduction on HIV and HCV seroconversion did not change substantially when analysis was restricted to the years after 1989 (i.e. when a methadone dose of ≥ 60 mg daily was more readily available for DU). Also, when the lower limit of adequate methadone dosage was adjusted to ≥ 80 mg daily, the effects of harm reduction on HIV and HCV seroconversion did not change substantially.

## DISCUSSION

Our data suggest that the combination of adequate methadone therapy and full participation in NEP contributed substantially to the reduction of the incidence of HIV and HCV in DU in Amsterdam, although a statistically significant effect was not seen when methadone dose or NEP were considered separately. It is likely that Amsterdam's comprehensive programme, in which methadone treatment and NEP are combined, explains the reported decline of HIV and HCV incidence.

We found no evidence that the effect of harm reduction was larger on HCV incidence than on HIV incidence, as our risk estimates for the different levels of harm reduction participation were comparable. One explanation might be that the Amsterdam harm reduction approach, which maintains contact with as many DU as possible, has an effect not only on injecting but also on sexual risk behaviour due to counselling and condom distribution. Our findings are in line with the reduction of sexual and drug-related risk behaviour seen in the ACS since the mid-1990s [7]. Having an HIV-positive steady partner was associated with a higher risk of HIV infection, showing that HIV is sexually transmitted more effectively than HCV.

The evaluation of HRPs is complicated because it is difficult to link participation in HRPs to outcome variables, such as the incidence of blood-borne infections. In some observational studies, methadone programmes and NEP have been shown to reduce the incidence of HIV but not HCV [5,6,15,16]. Ecological studies have shown a declining HCV prevalence after the introduction of NEP [17–20], while HCV incidence remained high. To our best knowledge, our study is the first that describes the combined effect of methadone therapy and NEP on HCV incidence, and over the longest period of time. The ACS among DU is a well-defined open cohort study, with ongoing recruitment, that has been followed over the past 20 years. On average, 90% of participants that visited the ACS a given calendar year returned the next year as well. Despite its great strengths, ACS is not a randomized controlled trial and therefore a causal association between harm reduction participation and risk for HIV or HCV infection cannot be proved. However, we could not think of any unmeasured confounder both affecting harm reduction participation and HIV or HCV infection.

Although NEP and methadone prescription were not available at the study setting, we cannot exclude that a cohort effect might explain partially the observed decrease in HIV and HCV incidence and injecting behaviour we observed in our cohort. Furthermore, risk behaviour was self-reported, and bias toward socially desirable answers could cause underestimation of the proportion engaged in risk behaviour. Although the data on HRP participation were also self-reported, Langendam *et al*. studied the harm reduction measures in the ACS and matched the self-reported methadone doses to the central methadone registry (CMR) and they found no clear difference in the self-reported dose and the dose at the CMR [21].

As expected, DU not injecting drugs in the past 6 months and taking a low dose of methadone daily (i.e. with limited dependence on harm reduction) and DU not injecting drugs in the past 6 months and not receiving any methadone (i.e. with no dependence on harm reduction) were at lower risk for HIV and HCV seroconversion than were DU fully participating in an HRP. Interestingly, the limited-dependence group were at lower risk for HIV and HCV seroconversion than the no-dependence group, although the difference was not statistically significant. It could be that, because DU receiving a low dose of methadone are still surrounded by the social–medical care network associated with the methadone therapy, they might return more easily to a higher dose of methadone or call for other help in case of problems than DU who have completely stopped methadone and are out of the network.

The most important implication of our study is that only when methadone is combined with provision of needles and syringes through exchange programmes is there a significant reduction of HIV and HCV incidence. Our finding is most important for countries with recent and sometimes explosive outbreaks of HIV and/or HCV among DU, as in the former Soviet Union and Asia [22,23]. To provide needles and syringes only or methadone only will not be sufficient to curb the rapid spread of these and other blood-borne infections among DU. It is essential to offer a comprehensive programme in which both measures are combined, preferably also with social–medical care and counselling.
